# The Streptococcal Protease SpeB Antagonizes the Biofilms of the Human Pathogen Staphylococcus aureus USA300 through Cleavage of the Staphylococcal SdrC Protein

**DOI:** 10.1128/JB.00008-20

**Published:** 2020-05-11

**Authors:** Katelyn E. Carothers, Zhong Liang, Jeffrey Mayfield, Deborah L. Donahue, Mijoon Lee, Bill Boggess, Victoria A. Ploplis, Francis J. Castellino, Shaun W. Lee

**Affiliations:** a Department of Biological Sciences, University of Notre Dame, Notre Dame, Indiana, USA; b Eck Institute for Global Health, University of Notre Dame, Notre Dame, Indiana, USA; c W. M. Keck Center for Transgene Research, University of Notre Dame, Notre Dame, Indiana, USA; d Department of Chemistry and Biochemistry, University of Notre Dame, Notre Dame, Indiana, USA; University of Illinois at Chicago

**Keywords:** SpeB, *Staphylococcus aureus*, *Streptococcus pyogenes*, adhesins, biofilms, proteases

## Abstract

Streptococcus pyogenes, or group A *Streptococcus* (GAS), is both a pathogen and an asymptomatic colonizer of human hosts and produces a large number of surface-expressed and secreted factors that contribute to a variety of infection outcomes. The GAS-secreted cysteine protease SpeB has been well studied for its effects on the human host; however, despite its broad proteolytic activity, studies on how this factor is utilized in polymicrobial environments are lacking. Here, we utilized various forms of SpeB protease to evaluate its antimicrobial and antibiofilm properties against the clinically important human colonizer Staphylococcus aureus, which occupies niches similar to those of GAS. For our investigation, we used a skin-tropic GAS strain, AP53CovS+, and its isogenic Δ*speB* mutant to compare the production and activity of native SpeB protease. We also generated active and inactive forms of recombinant purified SpeB for functional studies. We demonstrate that SpeB exhibits potent biofilm disruption activity at multiple stages of S. aureus biofilm formation. We hypothesized that the surface-expressed adhesin SdrC in S. aureus was cleaved by SpeB, which contributed to the observed biofilm disruption. Indeed, we found that SpeB cleaved recombinant SdrC *in vitro* and in the context of the full S. aureus biofilm. Our results suggest an understudied role for the broadly proteolytic SpeB as an important factor for GAS colonization and competition with other microorganisms in its niche.

**IMPORTANCE** Streptococcus pyogenes (GAS) causes a range of diseases in humans, ranging from mild to severe, and produces many virulence factors in order to be a successful pathogen. One factor produced by many GAS strains is the protease SpeB, which has been studied for its ability to cleave and degrade human proteins, an important factor in GAS pathogenesis. An understudied aspect of SpeB is the manner in which its broad proteolytic activity affects other microorganisms that co-occupy niches similar to that of GAS. The significance of the research reported herein is the demonstration that SpeB can degrade the biofilms of the human pathogen Staphylococcus aureus, which has important implications for how SpeB may be utilized by GAS to successfully compete in a polymicrobial environment.

## Abstract (precis)

Streptococcus pyogenes (GAS) causes a range of diseases in humans, ranging from mild to severe, and produces many virulence factors in order to be a successful pathogen. One factor produced by many GAS strains is the protease SpeB, which has been studied for its ability to cleave and degrade human proteins, an important factor in GAS pathogenesis. An understudied aspect of SpeB is the manner in which its broad proteolytic activity affects other microorganisms that co-occupy niches similar to that of GAS. The significance of the research reported herein is the demonstration that SpeB can degrade the biofilms of the human pathogen Staphylococcus aureus, which has important implications for how SpeB may be utilized by GAS to successfully compete in a polymicrobial environment.

## INTRODUCTION

Streptococcus pyogenes, or group A *Streptococcus* (GAS), is a species of Gram-positive bacteria and a common colonizer of human skin and mucosal surfaces (1–3). Asymptomatic carriage of this organism is prevalent, especially in young children, with 15 to 20% harboring GAS without apparent disease symptoms (3). It is also an exclusively human pathogen and the causative agent of common and self-limiting minor infections, such as pharyngitis (strep throat) and skin impetigo, which cause about 600 million and 100 million infections, respectively, annually (2). In rare cases, GAS can cause severe invasive disease, including necrotizing fasciitis and streptococcal toxic shock syndrome, through breach of the epithelial barrier and entry into the bloodstream (1–5).

GAS produces a large array of surface-expressed and secreted factors that contribute to its ability to survive in the host, cause disease, and invade deeper tissues. One extensively studied factor is SpeB (streptococcal pyrogenic exotoxin B), a cysteine protease with multiple proposed roles in GAS pathogenesis (6). The gene encoding SpeB is highly conserved across GAS strains (7–9), although expression and secretion of the SpeB protein are more variable. The SpeB enzyme is initially produced as an inactive 40-kDa zymogen (SpeBz) followed by autocatalytic cleavage to the 28-kDa active enzyme (SpeBm), a multistep process with several intermediates (10–12). Reduction of the cysteine-192 residue is also required for mature enzyme activity (12–14).

The contributions of SpeB to GAS pathogenesis have not been fully elucidated, but it has been shown to degrade multiple types of host proteins. Cleavage of extracellular matrix (ECM) and junction proteins is hypothesized to promote bacterial colonization and early invasion (15–17). SpeB has also been shown to degrade human immune system components, including immunoglobulins and chemokines associated with inflammatory and antibacterial responses (18–20). Regarding its pathogenicity, SpeB can degrade not only various host cell proteins but streptococcal proteins as well, including the plasminogen activator streptokinase (SK) (21) and streptococcal superantigens (22). Proteolytic cleavage of streptococcal proteins by SpeB is hypothesized to alter virulence and contribute to tissue tropism (15). An important consideration in many reported SpeB studies is the use of purified proteins and nonphysiological conditions. These limitations have been noted in studies demonstrating that SpeB does not cleave immunoglobulins under eukaryotic cell-like conditions and that the previously observed cleavage activity was unlikely to have functional consequences *in vivo* (23).

There is conflicting evidence regarding the role of SpeB in disease progression and pathogenesis. While several SpeB mutant studies show SpeB-dependent contributions to tissue damage, resistance to phagocytosis, and survival in mice (24–26), an inverse relationship between SpeB production and disease severity in human isolates of the M1T1 GAS strain has been observed (27). A more recent study showed that inactivation of the streptococcal regulator *srv* resulted in constitutive expression of SpeB, which in turn led to increased lesion size in mice, indicative of greater bacterial dissemination (28). Other studies have found no difference in virulence between SpeB mutants and wild-type GAS (29, 30). In a mouse model of invasive soft tissue infection, it was found that the hyaluronic acid capsule and surface-expressed M protein were critical for the observed pathology, but SpeB production did not contribute to pathology (29). Although SpeB has been widely studied in view of host pathogenesis with various conclusions, it is likely that strain- and context-dependent effects on the human host, as well as the infection system utilized for studies, play a major role in the effects of SpeB on disease outcomes.

SpeB is a cysteine family protease with functions similar to those of major secreted proteases of *Staphylococcus* species, including disruption of host immune factors and cell-cell junctions (31). Additionally, studies have observed the role of these bacterially secreted proteases in influencing polymicrobial dynamics. Previous investigations have shown that in competition for nasal mucosa between Staphylococcus aureus and Staphylococcus epidermidis, S. epidermidis strains that produce the serine protease Esp are able to outcompete and eradicate nasal colonization by S. aureus (32, 33). Compared to the large number of host-based studies of SpeB, little research has been conducted on potential roles of SpeB against other microorganisms in a polymicrobial environment. Another human pathogen and skin colonizer, Staphylococcus aureus, can occupy niches similar to those occupied by the S. pyogenes strain AP53CovS+, a skin-tropic, noninvasive GAS strain (34, 35). S. aureus is clinically important due to its ability to cause human disease in the form of skin infections, sepsis, and pneumonia (36–39). Though both organisms have been isolated from impetigo lesions (40), the mixed microbial dynamics between S. pyogenes and S. aureus have been largely unexplored, and they may provide a model by which to investigate alternative functions of known host-acting virulence factors such as SpeB.

A clinically important phenotype of S. aureus is its ability to form biofilms, including on abiotic surfaces such as implants and medical devices (41). However, to our knowledge there have not been investigations into antimicrobial or antibiofilm properties of the SpeB protease. We hypothesized that the proteolytic activity of SpeB would disrupt the normal growth and biofilm formation of S. aureus. In our investigation, we observed a SpeB- and dose-dependent degradation of the biofilm in S. aureus strain USA300, though planktonic growth was not abrogated. We hypothesized that SpeB could disrupt S. aureus biofilms by targeting cell wall-anchored proteins, and our results showed that the SdrC adhesin, important to S. aureus biofilm formation on abiotic surfaces (42, 43), is directly cleaved by SpeB both in a recombinant form and directly from S. aureus cells in a biofilm. Our results suggest a role for SpeB as an important factor for GAS colonization and competition with other microorganisms in its niche.

## RESULTS

### Characterization of AP53CovS+ and an isogenic Δ*speB* mutant.

AP53CovS+ is an M53 GAS strain belonging to the *emm* pattern D subgroup (34), members of which are skin-tropic and have higher levels of SpeB secretion than strains with *emm* patterns A to C (21). To evaluate the effects of SpeB production by the GAS strain AP53CovS+, we first generated an isogenic *speB* deletion mutant. Importantly, growth remained unchanged in the absence of the *speB* gene (see Fig. S1A in the supplemental material). To determine changes in proteolytic activity in the SpeB knockout, stationary-phase culture supernatants of the GAS strains were incubated with the general proteolytic substrate azocasein. A reducing buffer with dithiothreitol (DTT) was added to the reaction mixture to ensure reduction of the cysteine-192 (Cys^192^) residue essential for proteolytic activity in the AP53CovS+ (wild-type) strain. As shown in Fig. S1B, the wild-type AP53CovS+ strain had a higher secreted proteolytic activity than its isogenic *speB* deletion mutant, as measured by azocasein digestion. This was also demonstrated by zones of clearing around GAS colonies in milk agar, as the Δ*speB* strain has a reduced zone of clearing compared to its wild-type counterpart (see Fig. S1C).

### Purification of recombinant SpeB protein.

Active recombinant SpeB protease (r-SpeB) was purified for its use in antimicrobial and antibiofilm studies with S. aureus. To reduce issues with insolubility during purification, the *speB* gene was cloned without its signal sequence, and a His tag was added to the C terminus of its product, as this region remains after cleavage of the proenzyme form of SpeB (10). A product of ∼28 kDa was observed after purification on nickel-nitrilotriacetic acid (Ni-NTA). This corresponded to the size of the active form of SpeB, demonstrating that autocatalysis occurred during the purification process, as the 40-kDa zymogen product was not observed (see Fig. S2). Following dialysis against phosphate buffer, purified SpeB was shown to have proteolytic activity in the azocasein digestion assay (see Fig. S3). The catalytically inactive SpeB mutant, SpeB[C^192^S], was also purified on Ni-NTA, resulting in an approximately 40-kDa product (data not shown). This corresponds to the size of SpeB in its zymogen form, demonstrating that the mutant protein does not have catalytic activity for cleavage into its active form. SpeB[C^192^S] was used as an enzymatic activity control in experiments with r-SpeB.

### SpeB does not contribute to host cell cytotoxicity.

We first assessed whether the active r-SpeB was cytotoxic to a line of human keratinocytes (HaCaTs). The human cells were directly incubated with r-SpeB at a range of concentrations, and cytotoxicity was assayed by ethidium homodimer uptake after 6 h to assess membrane permeabilization. Cytotoxicity levels above vehicle control were not observed for any of the tested concentrations of r-SpeB (see Fig. S4). We next performed direct infection studies with the GAS strains AP53CovS+ and AP53CovS+ Δ*speB* on HaCaTs for 6 h at a multiplicity of infection (MOI) of 10. While cytotoxicity levels above control were observed for GAS infection overall, there was no significant difference in cytotoxicity between the SpeB-producing and -deficient GAS strains (see Fig. S5). Together, these data show that SpeB production by the skin-tropic GAS strain AP53CovS+ does not have a direct effect on *in vitro* cytotoxicity.

### Recombinant SpeB does not impact normal planktonic growth of S. aureus USA300.

**FIG 1:**
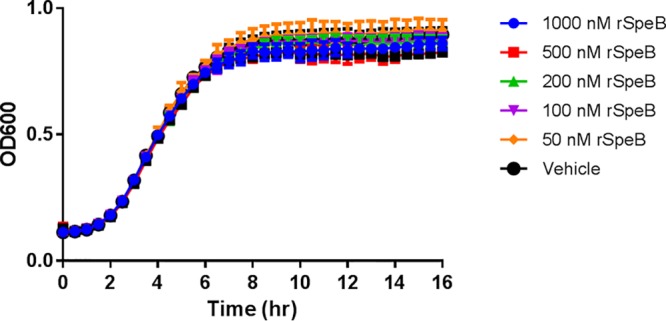
Growth curve of S. aureus USA300 in the presence of active recombinant SpeB and a vehicle control of phosphate buffer over 16 h.

As SpeB is a cysteine protease with broad activity and multiple host and streptococcal protein targets, we aimed to assess whether its proteolytic activity would affect the viability of another bacterial pathogen: S. aureus USA300. Overnight USA300 cultures were diluted in tryptic soy broth (TSB) and optimized for planktonic growth, whereupon purified r-SpeB was added at concentrations ranging from 1,000 nM to 50 nM. Phosphate-buffered saline (PBS) was used as a vehicle control. Growth was measured by determining optical density at 600 nm (OD_600_) every 30 min for 16 h. As shown in Fig. 1, all USA300 cultures exhibited similar sigmoidal growth patterns regardless of the concentration of r-SpeB present. These data show that recombinant SpeB does not have bacteriostatic or bactericidal effects on S. aureus USA300, as its normal planktonic growth was not slowed or abrogated.

### Recombinant SpeB- and SpeB-secreting GAS culture supernatants exert effects on S. aureus biofilms.

**FIG 2:**
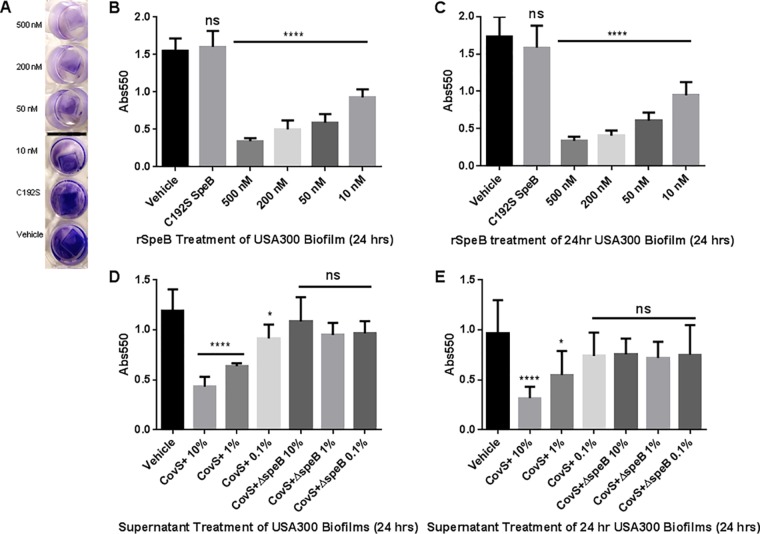
Biofilm density after SpeB treatment measured by crystal violet assay. (A) Representative image of dose-dependent reduction in biofilm density after treatment with r-SpeB. (B) Crystal violet assay of biofilm formation over 24 h with r-SpeB. (C) Crystal violet assay of the degradation of a 24-h-old biofilm after an additional 24-h treatment with r-SpeB. (D) Biofilm formation over 24 h in the presence of supernatants of AP53CovS+ and AP53CovS+ Δ*speB* GAS strains. (E) Biofilm degradation of 24-h-old biofilm over an additional 24 h with GAS supernatants. *, *P* < 0.05; ****, *P* < 0.0001; ns, not significant.

While we did not observe direct antimicrobial activity by SpeB in the form of growth inhibition, we considered the host proteins targeted by SpeB, the degradation of which would not directly result in cell death. We hypothesized that SpeB antagonized competing microbes by other means, including manipulation of biofilms. We prepared biofilms by growing USA300 cultures in TSB-NaCl-glucose to promote biofilm formation. Biofilms were grown on polyvinyl chloride (PVC) coverslips, as PVC is a medically relevant plastic surface and is not cell culture treated, thus avoiding “false positives” for biofilm formation that may occur on cell culture-treated surfaces. To more broadly assess the impact of SpeB on S. aureus biofilms, both biofilm formation and biofilm degradation were assessed. The impact on biofilm formation was determined by adding treatments to USA300 overnight cultures, diluted 1:100, at the beginning of biofilm formation. For biofilm degradation, treatments were added to biofilms that had previously formed untreated for 24 h. For both formation and degradation experiments, biofilms were treated for 24 h before a crystal violet assay was used as a readout of biofilm density. We used active recombinant SpeB and the SpeB[C^192^S] catalytic mutant as biofilm treatments for precise control of the amount of protease added. A representative image of biofilms treated with r-SpeB and assayed with crystal violet is shown in Fig. 2A. As shown in Fig. 2B and C, there is a dose-dependent reduction in biofilm density when biofilms are treated with active r-SpeB. Use of the SpeB[C^192^S] mutant at the highest concentration of r-SpeB (500 nM) did not affect S. aureus biofilm density, demonstrating that catalytic activity of SpeB is required for the observed biofilm disruption. Dose-dependent reduction was shown for both biofilm formation (Fig. 2B) and biofilm degradation (Fig. 2C). This shows that SpeB disrupts both early- and late-stage biofilms *in vitro*.

Based on our results obtained with r-SpeB, we hypothesized that native SpeB protease secreted from GAS would also antagonize S. aureus biofilms. Stationary-phase culture supernatants of GAS strain AP53CovS+ and its isogenic Δ*speB* mutant were used for treatment of the USA300 biofilms. Briefly, the GAS strains were grown to stationary phase in THY broth (Todd-Hewitt broth supplemented with yeast extract), and the supernatants were applied to the biofilms as percent volumes of the TSB+ (TSB supplemented with 1% NaCl and 0.5% glucose) used for biofilm growth. THY broth was used as the vehicle control. A dose-dependent reduction in biofilm density was observed according to the percent volume of AP53CovS+ supernatant present for both biofilm formation and degradation (Fig. 2D and E). This same dose dependency was not observed with supernatants from the isogenic Δ*speB* mutant, where levels of biofilm density were similar to the vehicle control.

**FIG 3:**
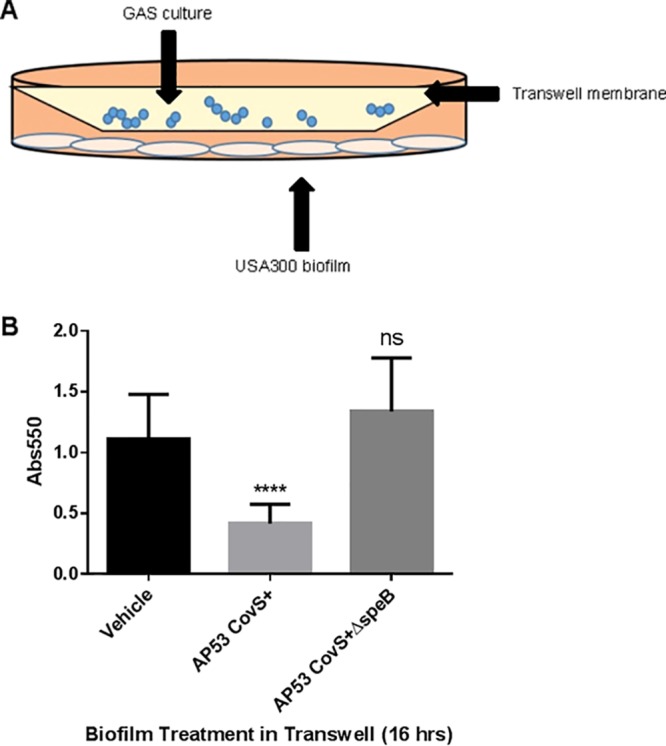
(A) Schematic of Transwell assay for biofilm degradation by GAS cultures. (B) Crystal violet assay of USA300 biofilms after 16 h incubation in a Transwell with GAS cultures (AP53CovS+ and AP53CovS+ Δ*speB*) and THY medium vehicle. ****, *P* < 0.0001; ns, not significant.

We utilized a coculture model with both GAS bacteria and USA300 biofilms in a Transwell system to assess any biofilm-antagonizing effects by actively growing GAS cultures (Fig. 3A). USA300 biofilms were grown untreated on PVC coverslips before being placed below a Transwell with a 0.4-μm membrane (Corning), to allow the passage of secreted factors but not bacteria. AP53CovS+ and AP53CovS+ Δ*speB* GAS cultures were grown above the membrane. THY medium was used for optimal SpeB production by GAS, and we previously established that growth in THY alone did not disrupt USA300 biofilms (data not shown). As shown in Fig. 3B, there was a SpeB-dependent, contact-independent disruption of USA300 biofilms by GAS cultures after 16 h.

**FIG 4:**
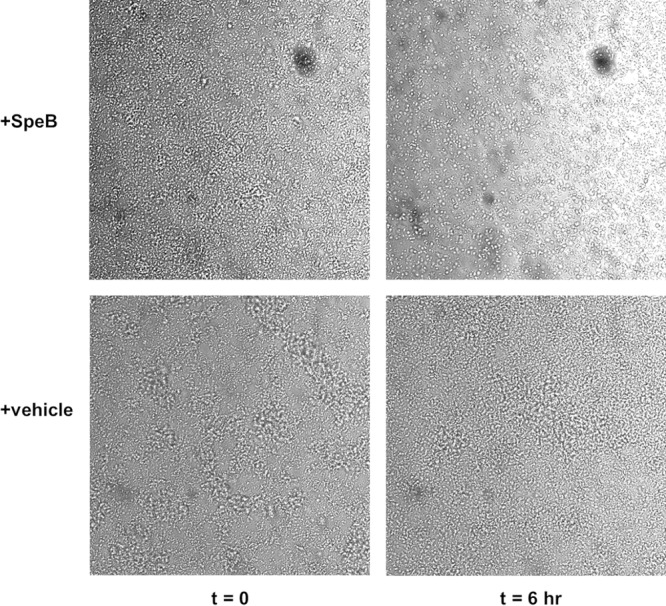
USA300 biofilms treated with 100 nM r-SpeB (top) or PBS vehicle control (bottom) at time zero (left) and 6 h (right). Images were taken on a Nikon Ti-E inverted microscope and processed on ImageJ.

In order to visualize biofilm disruption by SpeB, biofilms were grown in imaging dishes for 24 h before being visualized on a Ti-E inverted microscope (Nikon) for 6 h with r-SpeB or vehicle treatment. By 6 h, there was visible disruption of S. aureus microcolonies into individual cocci in the presence of SpeB, whereas this morphology was not seen in S. aureus cultures not treated with active r-SpeB (Fig. 4; also, see Videos S1 and S2 in the supplemental material).

To assess whether SpeB-mediated cleavage of S. aureus biofilms occurred on host surfaces, biofilms were grown on confluent monolayers of HaCaTs fixed in 4% paraformaldehyde. As in our previous biofilm studies with endogenous SpeB, GAS culture supernatants were added to the S. aureus cultures prior to biofilm formation over 24 h. A significant reduction in the biofilm was observed when it was treated with AP53CovS+ supernatants compared to treatment with Δ*speB* mutant supernatant or no treatment (see Fig. S6).

Taken together, these data show that SpeB, in both recombinant and natively produced forms, has demonstrable antibiofilm activity against S. aureus USA300, both on early- and late-stage biofilms and under different nutrient conditions.

We next sought to determine the mechanism by which SpeB acts to disrupt S. aureus biofilms. As previously described, SpeB is a protease with multiple defined targets. While it is likely that SpeB may target multiple proteins in order to cause the observed biofilm disruption of S. aureus, we were particularly interested in cell wall-associated proteins that were nonessential for survival. Given our finding that SpeB did not have antimicrobial activity, the proteins on the surface of S. aureus would need to be accessible to the protease. SdrC (serine-aspartate repeat protein C) is a cell wall-anchored adhesin that has previously been associated with S. aureus biofilm formation (44), more specifically in attachment to plastic surfaces and cell-cell adhesion (42, 43). A deletion of the *sdrC* gene has been shown to decrease biofilm density, as measured by crystal violet assay (42). In order to evaluate whether SpeB has activity against SdrC, we cloned and purified the A region of the SdrC protein (45) with an N-terminal His tag (see Fig. S7). Our SdrC region A His tag protein allows full accessibility of the A region on the cell wall for our studies and showed increased stability over purification of the full-length SdrC protein (data not shown).

### SpeB cleaves and degrades SdrC region A *in vitro*.

**FIG 5:**
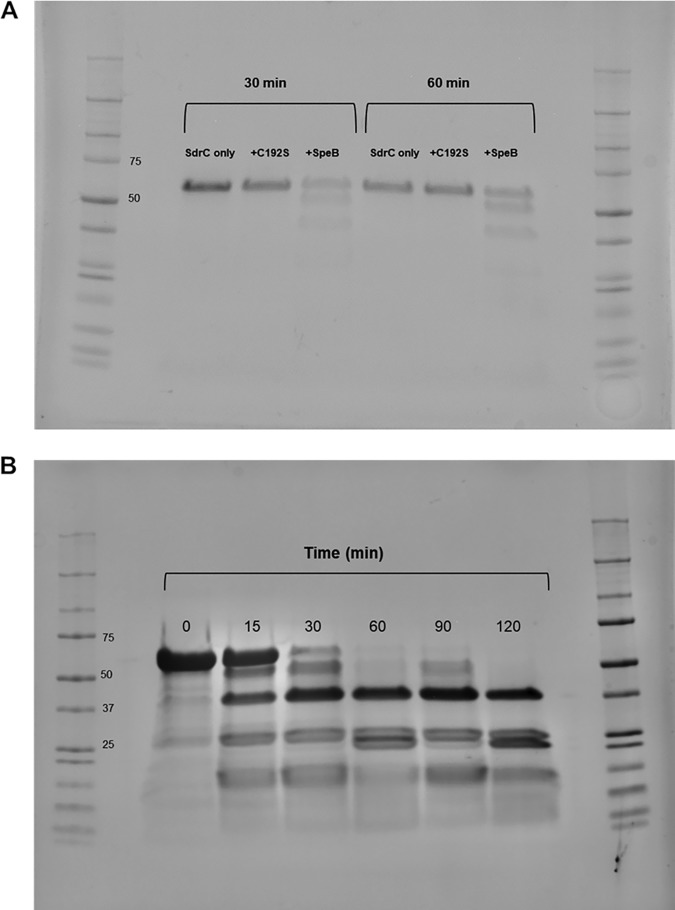
*In vitro* incubation of recombinant SpeB with recombinant SdrC region A. (A) Incubation of SdrC and SpeB[C^192^S]-vehicle at a 10:1 ratio for 30 and 60 min. (B) Incubation of SdrC and SpeB at a 100:1 ratio for 0 to 120 min. Protein products were run on SDS-PAGE and Coomassie stained prior to imaging.

**FIG 6:**
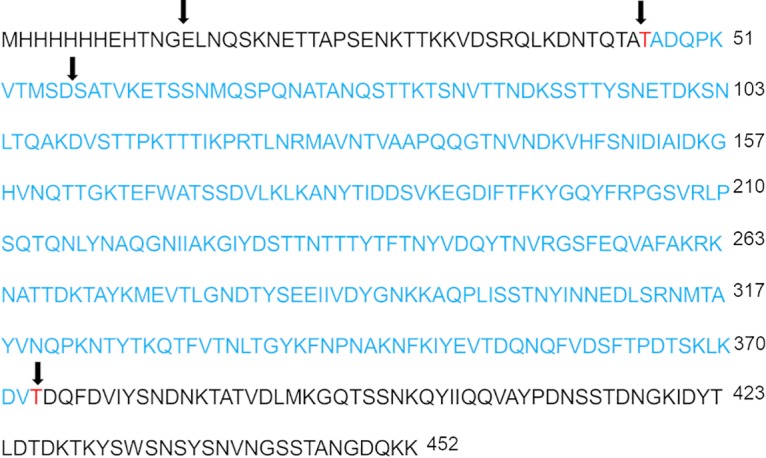
Sequence of SdrC region A with an N-terminal His tag with predicted cleavage sites of SdrC region A after *in vitro* incubation with r-SpeB. Reactions were assessed by MALDI and LC/MS after 15 min of incubation. Cleavage sites are shown with arrows. An approximately 36-kDa product seen to be stable over the incubation time course (shown in blue) was predicted to result from cleavage on either side of the threonine residues shown in red.

We first evaluated if purified recombinant SpeB is capable of cleavage activity against SdrC. SdrC-A and r-SpeB or r-SpeB[C^192^S] were first incubated at 37°C in phosphate buffer at a 10:1 ratio, respectively, for 30 and 60 min. SdrC treated with SpeB showed degradation products within 30 min, while SdrC treated with inactive enzyme or left untreated remained stable (Fig. 5A; also, see Fig. S8). Next, the ratio of SdrC to SpeB was changed to 100:1, and the time course was expanded to 2 h. Recombinant SpeB rapidly cleaved the SdrC parent product into multiple fragments, with a highly stable fragment around 36 kDa (Fig. 5B). To determine the cleavage specificity of SpeB, we incubated SdrC and SpeB for shorter times than our previous time course (<15 min) and subjected the fragments to matrix-assisted laser desorption ionization (MALDI) and liquid chromatography-mass spectrometry (LC/MS) analysis. From the identified peptide fragments, we used Sequence Editor software to predict cleavage sites within the recombinant SdrC-A protein, including the N-terminal histidine tag. As shown in Fig. 6, predicted cleavage sites that would result in the previously observed stable ∼36-kDa product (shown in blue) were residues 46 to 372 or residues 47 to 373, with potential cleavage on either side of a threonine residue (shown in red).

### Biofilm incubation with SpeB results in cleaved SdrC products.

**FIG 7:**
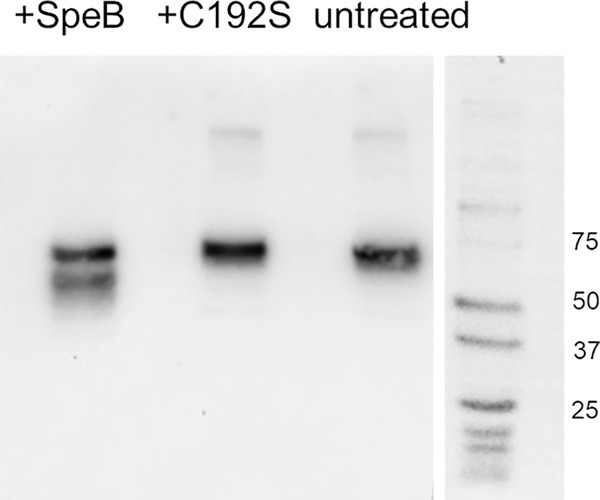
Western blot of SdrC in supernatants of USA300 biofilms. Biofilms were treated with SpeB or SpeB[C^192^S] or left untreated for 2 h, after which the supernatants were collected. Rabbit serum antibody against SdrC region A was used at a 1:1,000 ratio to detect SdrC fragments in the supernatant. The top band present in all three conditions runs at approximately 75 kDa, and the cleaved products in the SpeB condition run at approximately 55 to 60 kDa.

While we observed rapid degradation of the r-SdrC region A by r-SpeB, we sought to demonstrate if a similar phenomenon would be observed in a whole-biofilm model. USA300 biofilms were grown on PVC coverslips for 24 h, then washed with PBS, and treated with r-SpeB, r-SpeB[C^192^S], or buffer for 2 h. Supernatants, containing any cleaved proteins, were removed, pooled, filtered, and concentrated. Protein concentrations were normalized by *A*_280_ and were used for sodium dodecyl sulfate-polyacrylamide gel electrophoresis (SDS-PAGE) and Western blot analysis. Rabbit serum antibody against SdrC was used to detect any reactive SdrC fragments in the supernatant. As shown in Fig. 7, a product of approximately 75 kDa was detected with anti-SdrC antibody under all three conditions. However, a population of lower-molecular-weight products between 50 and 60 kDa was observed only under conditions in which the biofilm was incubated with SpeB. These products are larger than the approximate 50-kDa size of our recombinant SdrC region A, suggesting that cleavage of SdrC from the biofilm occurs in the B-repeat regions downstream of the A region (43). These data demonstrate that cleavage products of SdrC that are unique to the presence of SpeB can be observed in the context of the full S. aureus biofilm and correlate with the disruption of biofilms observed only under conditions where SpeB is present.

## DISCUSSION

Group A *Streptococcus* produces a myriad of virulence factors that enable it to be both a successful pathogen and asymptomatic colonizer of its human host. The secreted cysteine protease, SpeB, has been widely studied for its effects on host proteins and its contributions to pathogenesis, but little attention has been given to how SpeB may be utilized in polymicrobial dynamics. We hypothesized that the broad proteolytic activity of SpeB may be used by GAS to compete in a polymicrobial environment. To begin investigating this hypothesis in the skin-tropic, noninvasive strain of group A *Streptococcus*, AP53CovS+, we tested whether SpeB impacted the growth and the biofilm development and maintenance of the human pathogen and skin colonizer Staphylococcus aureus. Our results showed SpeB-dependent reduction in the formation and maintenance of the S. aureus biofilm, observed with both recombinant and endogenous SpeB (Fig. 2 and 3). In our investigation into the possible mechanisms of SpeB-dependent biofilm disruption, we hypothesized that SpeB might target SdrC, a serine-aspartate repeat protein expressed on the surface of S. aureus linked to biofilm formation. Our findings show that recombinant SdrC can be directly cleaved by SpeB (Fig. 5); furthermore, we show that native SpeB produced by GAS generates specific cleavage products of SdrC concomitant with biofilm dispersal of S. aureus (Fig. 6).

Many excellent studies and reviews have summarized the role of bacterial proteases in host cell infection and colonization, but less focus has been given to the role of secreted proteases in polymicrobial dynamics, including how they may be used in an antimicrobial or antibiofilm manner to kill or disperse competing microbes. While our results did not show that SpeB has direct antimicrobial activity against S. aureus USA300 (Fig. 1), we considered that SpeB is known to cleave surface proteins on GAS itself (46) and hypothesized that this activity could be extended to other microbial pathogens, impacting properties such as biofilm formation without direct killing. Previous studies showed that Esp, a secreted serine protease of Staphylococcus epidermidis, degraded S. aureus biofilms by targeting the biofilm matrix and cell wall proteins and further inhibited nasal colonization by S. aureus by targeting surface host receptors (32). Similar to our results with SpeB, Esp alone did not have demonstrable bactericidal activity against S. aureus but did result in increased susceptibility of S. aureus to host immune components (47). It is reasonable to consider that SpeB-mediated disassembly of S. aureus biofilms increases the susceptibility of S. aureus to antimicrobial agents or overall bacterial clearing, but determining the extent of this activity and identifying possible synergistic effects with antimicrobials require further investigation.

In our investigation into the possible mechanisms of our observed SpeB-dependent biofilm disruption, we hypothesized a role for SdrC, a serine-aspartate repeat protein expressed on the surface of S. aureus. Previous studies showed that SdrC promotes cell-cell adhesion in S. aureus through homophilic interactions and mediates attachment to hydrophobic abiotic surfaces (43). The N terminus of SdrC contains an A region with three subdomains (N1, N2, and N3). It has been shown that using a peptide to block the N2 and N3 subdomains reduced biofilm formation of S. aureus (43). Similarly, Barbu et al. reported that a knockout of the *sdrC* gene in S. aureus had reduced biofilm formation and that binding sites within the N2 subdomain promoted biofilm formation (42). Based on these previous studies, we hypothesized that the SpeB-dependent biofilm disruption could be due to disruption of SdrC region A by SpeB. Incubation of our SdrC-A product with recombinant SpeB *in vitro* at a 100:1 ratio rapidly resulted in a series of cleavage products, while SdrC-A remained stable when incubated with the SpeB[C^192^S] mutant or left untreated. MALDI and LC/MS analysis of lower-molecular-weight cleavage products resulted in several predicted cleavage sites, including those at Thr residues that resulted in a more stable cleaved product of SdrC (Fig. 6). SpeB has not been shown to have high substrate sequence specificity and generally has been shown to have cleavage preference for three amino acids upstream of the cleavage site and a hydrophobic residue preferred at the P2 position before the cleavage site (15, 48). From our analysis of the cleaved SdrC fragments, we speculate that early cleavage from the N terminus of SdrC occurs first, with overlap between subsequent cleavage products, which suggests that independent rather than sequential cleavage of SdrC by SpeB likely occurs. These *in vitro* cleavage products are likely dependent on SpeB concentration, as earlier *in vitro* incubation at a 10:1 ratio resulted in few products below 36 kDa, indicative of their rapid degradation at higher SpeB concentrations.

While *in vitro* incubation demonstrated that SpeB was capable of rapidly cleaving SdrC region A, it was necessary to assess if this activity occurred within whole S. aureus biofilms and contributed to the observed biofilm disruption. For our whole-biofilm incubation studies, rabbit serum anti-SdrC antibody was used to blot for SdrC products in the supernatants of SpeB-treated SdrC biofilms. Interestingly, an SdrC product of approximately 75 kDa was present under all conditions. While not the size of full-length SdrC, this does correspond to the predicted size of both region A and the B repeats preceding the Ser-Asp repeat regions (43), suggesting that some portion of SdrC is present in the biofilm supernatants even in the absence of SpeB. Importantly, a population of lower-molecular-weight products was observed only in the supernatants of biofilms that had been incubated with active SpeB (Fig. 7). This population of cleaved products was larger than the approximate 50-kDa size of SdrC region A. This suggests that in the context of a whole biofilm, SpeB may cleave SdrC within the B-repeat region prior to further processing, resulting in the products larger than the 50 kDa observed. Further studies are required to more fully elucidate the mechanism of SpeB-dependent disruptions of S. aureus biofilms.

In this study, we have demonstrated that SpeB is a potent disruptor of S. aureus biofilms and that this phenotype is at least in part due to cleavage of the SdrC adhesin. Disruption of bacterial biofilms by proteases has been studied previously, with protease origins ranging from bacteria to fungi to plants (32, 49, 50). While not inherently antimicrobial, disruption of biofilms can render bacteria more susceptible to antimicrobial agents and immune components. Antibiofilm activity, to our knowledge, has not previously been attributed to SpeB, and our data have implications for how it may be utilized by GAS to compete in a polymicrobial environment. While the cleavage of SdrC is likely an important factor in the SpeB-dependent biofilm disruption, based on the known broad activity of SpeB, it is highly likely that there are other components in both the cell wall and matrix that may be targeted. It will also be important to assess if the antibiofilm activity of SpeB extends more broadly to other organisms in the colonizing niche of GAS. It is interesting that r-SpeB alone, at the concentrations tested, did not have demonstrable antimicrobial activity against S. aureus or cytotoxic activity against human keratinocytes and that GAS infections with our AP53CovS+ strains did not result in SpeB-dependent cytotoxicity *in vitro*. These data suggest that SpeB exhibits low direct toxicity but has an important role in microenvironment manipulation by GAS, whether through cleavage of host factors or antagonism of biofilms. Overall, our studies suggest that in the skin-tropic, noninvasive GAS strain AP53CovS+, SpeB allows GAS to manipulate its microenvironment through biofilm antagonism, and our study provides support for the understudied role of secreted proteases in polymicrobial dynamics and microbiome stability.

## MATERIALS AND METHODS

### Bacterial growth conditions.

Unless otherwise specified, GAS strains AP53CovS+ and AP53CovS+ Δ*speB* were grown in Todd-Hewitt broth supplemented with 2% yeast extract (THY) at 37°C. S. aureus USA300 was grown in tryptic soy broth (TSB) at 37°C. For biofilm experiments, USA300 was grown in TSB supplemented with 1% NaCl and 0.5% glucose (TSB+) (see “Biofilm assays” below). For visualization of casein digestion, colonies of GAS were stabbed into 10% skim milk Columbia agar plates and incubated overnight at 37°C.

### *speB* gene deletion.

The GAS strain AP53 was described by Berge and Sjöbring in 1993 (51), and its whole-genome sequence was determined in 2016 (34). In 2013, it was discovered that the original clinical isolate contained a mutated two-component signal protein (CovS) gene, which was not expressed (AP53CovS−) (35). This gene was mutated to the WT-CovS gene (AP53CovS+), and the resulting strain was used as the GAS line for all studies, as it expresses higher levels of SpeB than the CovS− strain (35).

### Generation of a targeted deletion of the *speB* gene.

To construct the targeting vector (TV) for the *speB* (streptococcal pyrogenic exotoxin B gene) deletion in AP53CovS+ cells, a fragment was amplified by PCR as the insert for the TV spanning 538 bp upstream of the ATG signal peptide for *speB* and 435 bp downstream of the TAG stop codon for *speB*. During this process, the restriction endonuclease sites 5′-NotI and 3′-XhoI were also cloned into the two ends of the insert DNA by PCR primers, and they were used for insertion into the same sites of the temperature-sensitive plasmid pHY304 (from M. J. Walker, Queensland, Australia), which also contained the erythromycin resistance gene (*erm*) in the plasmid backbone. The primers used were SpeBKONot5F, SpeBKONot5R, SpeBKOXho5F, and SpeKOXho3R (see Table S1 in the supplemental material).

The resulting targeting plasmid was then transformed into AP53CovS+ cells by electroporation. Chromosomal integration via allelic replacement was achieved by single crossover (SCO) at 30°C for plasmid replication and then switched to 37°C overnight on an erythromycin (Erm) plate (Erm, 3 μg/ml). Surviving colonies were checked by PCR for *erm* gene insertion. A similar procedure for a secondary crossover, or double crossover (DCO), was performed in order to remove the *speB* gene and the targeting vector backbone with the insert in TV. In brief, SCO cells were grown at 30°C for 2 h and plated on a THY plate without Erm to 37°C overnight. Colonies selected were plated on both THY plates and THY-Erm plates, and colonies that grew in THY but not THY-Erm were selected due to the loss of the *erm* gene after DCO. For confirmation of *speB* gene inactivation, positive colonies were screened by PCR using primers upstream and downstream of the *speB* gene (SpeBext5F and SpeBext3R [see Table S1]). The final GAS cell line with the *speB* gene deleted is referred to as AP53CovS+ Δ*speB*.

### Plasmid constructions for protein expression. (i) Generation of the SpeB expression construct.

An expression construct was created in order to generate recombinant SpeB containing a C-terminal histidine tag and with the signal peptide, consisting of the first 81 nucleotides, removed. Primers (SpeB-F and SpeB-R) were used to insert restriction sites for NdeI and EcoRI and a 6×His tag. The PCR product from these primers was digested and ligated into the pET42A expression plasmid. Clones generated from BL21 cell transformation were sequenced to confirm the correct insertion. The expression construct was named pET42aSpeB.

### Generation of catalytically inactive SpeB protein variants.

In SpeB, the cysteine residue at position 192 is essential for enzymatic activity (10). We generated a catalytically inactive variant of the SpeB protein by replacing C192 with serine (SpeB[C^192^S]), using a series of PCR primers designated P1 to P4 (see Table S1). For this construct, two PCR steps were employed. The first PCR step added the NdeI restriction site in front of SpeB residue D28 (primer P1) and amplified to the A198 residue with the C^192^S mutation (TGT to TCT) in the reverse primer (P2). A second PCR fragment was generated from SpeB residue Q186 with C^192^S in the 5′ primer (P3) to the end of SpeB protein at residue P398, followed by a 6×His tag and a stop codon (TGA); then, an EcoRI restriction endonuclease site was incorporated using the 3′ primer (P4). The second PCR step used the two products from first PCR as the DNA template for PCR amplification by primers P1 and P4, for a DNA fragment with a C^192^S mutation and NdeI and EcoRI in the 5′ and 3′ ends, respectively. This full-length C^192^S mutant DNA fragment was cloned into the pCRII-TOPO vector, confirmed by sequencing, digested with NdeI and EcoRI, and isolated for ligation into the same sites of the Escherichia coli expression vector pET42a. This pET42[C^192^S] expression vector was transfected and expressed in BL21(DE3) cells. The recombinant SpeB[C^192^S] protein was purified on a Ni-NTA column by routine procedures.

### (iii) Generation of the 6×His–SdrC-A expression construct.

To generate an expression construct for SdrC region A with an N-terminal His tag, the FastCloning technique was used as described previously (52). In brief, SdrC region A (amino acids 52 to 496, described previously [45]) was PCR amplified from S. aureus USA300 using primers pET15_sdrC-F and pET15_sdrC-A-R. The pET15 vector was amplified using primers OMF512 and OMF513 (see Table S1), a kind gift from the P. Champion group (University of Notre Dame). The insert and vector amplicons had a 16-bp overlapping region. The insert and vector PCR products were transformed into DH5α cells, and ampicillin (50 μg/ml) was used for selection. Purified plasmid was sequenced to confirm the insert, and the construct was transformed into BL21 cells for protein purification. The expression construct was named pET15SdrC-A.

### Purification of recombinant proteins.

For purification of recombinant SpeB (r-SpeB), recombinant SpeB[C^192^S] (r-SpeB[C^192^S]), and recombinant SdrC-A (r-SdrC-A), BL21 cells containing pET42aSpeB, pET42a[C^192^S], or pET15SdrC-A were grown under kanamycin (pET42aSpeB and pET42a[C^192^S]) or ampicillin (pET15SdrC-A) selection in Luria-Bertani (LB) medium, scaled up to 50 ml, and then scaled up to 1 liter in Terrific Broth (53) under shaking conditions at 37°C. When the culture reached an OD_600_ of 0.6 to 0.8, 100 μM IPTG (isopropyl-β-d-thiogalactopyranoside) was added for induction, and the culture was grown overnight at 25°C. Following induction, the bacteria were pelleted, collected, and resuspended in lysis buffer (50 mM NaH_2_PO_4_, 300 mM NaCl, 10 mM imidazole; pH 8.0) and spun again at 10,000 rpm for 10 min. The supernatant was removed, and the pellet was frozen at −80°C until lysis. Prior to lysis, the pellet was thawed on ice and resuspended in lysis buffer with 1 mg/ml lysozyme and protease inhibitor cocktail (Sigma). The pellet was incubated at 37°C for 30 min, with 25 U/ml universal nuclease (Pierce) added during the last 10 min. The lysate was then sonicated for 10 min with the following conditions: 20% amplitude with 2-s pulses and 1-s rest. The lysate was spun at 12,500 rpm for 40 min, and the soluble fraction was removed and filtered. Proteins were purified using a Ni-NTA column on an Äkta Pure (GE). Elution fractions were concentrated 1:10 with a filter with a molecular weight cutoff of 10,000 and dialyzed against phosphate-buffered saline overnight at 4°C. Proteins were confirmed by SDS-PAGE, MALDI, and MS/MS analysis. Proteolytic activity of r-SpeB was confirmed by azocasein digest as previously described, with modifications (54). Briefly, purified proteins from the elution fraction were incubated 1:1 with activation buffer (0.1 M sodium acetate [NaAc], 1 mM EDTA, 1 mM DTT; pH 5.0) at 37°C for 30 min before incubation with azocasein reagent (2% azocasein in 0.1 M NaAc, 1 mM EDTA; pH 5.0) 1:1 at 37°C for 30 min. The reaction was quenched with 6% trichloroacetic acid (TCA), and the precipitate was spun down at 10,000 × *g* for 10 min. The absorbance of the supernatant was read at 366 nm.

### Growth curves.

Overnight cultures of S. aureus USA300 were diluted to an OD_600_ of 0.1 in fresh THY medium. Dilute cultures were mixed with various concentrations of r-SpeB and plated on a 96-well microtiter plate. The plate was incubated at 37°C, and OD_600_ readings were taken every 30 min for 16 h using the Synergy H1 microplate reader (BioTek) to measure the growth of planktonic USA300 cultures in the presence of r-SpeB.

### Host cell cytotoxicity using an ethidium homodimer assay.

Human keratinocytes (HaCaTs) were grown to 90% confluence in Dulbecco’s modified Eagle medium (DMEM) supplemented with 10% fetal bovine serum (FBS). For infection studies, overnight cultures of AP53CovS+ and AP53CovS+ Δ*speB* were added to the HaCaTs at a multiplicity of infection (MOI) of 10 bacteria per host cell, and the HaCaTs were infected for 6 h at 37°C and 5% CO_2_. To evaluate cytotoxicity of recombinant protease alone, r-SpeB was added to the HaCaTs and incubated for 6 h at 37°C and 5% CO_2_. At the conclusion of the incubation with bacteria or recombinant SpeB, HaCaTs were washed with PBS, and then a 4 μM concentration ethidium homodimer solution in PBS was applied to the cells for 30 min in the dark. A fluorescent reading was taken on a microtiter plate reader (BioTek) at an excitation of 528 nm and an emission of 617 nm. To normalize for variation in the number of cells per well, a 0.1% solution of saponin was added to permeabilize all cells, and a second fluorescence reading was taken with the same settings. Percent membrane permeabilization was calculated as the first fluorescence reading divided by the second reading.

### Biofilm assays.

Prior to biofilm formation, PVC coverslips (Fisher) were sterilized under UV light in 12- or 6-well cell culture-treated plates. Single colonies of USA300 grown on TSB agar plates were grown overnight in TSB and then diluted 1:100 in TSB+. Diluted USA300 cultures were added to the wells on top of the PVC coverslips.

### (i) Biofilm studies with recombinant SpeB protease.

For biofilm formation assays with r-SpeB, r-SpeB and the SpeB[C^192^S] inactive variant were added at specified concentrations to the diluted USA300 cultures prior to application on the PVC coverslips. Biofilms were left to form in the presence of r-SpeB for 24 h. For biofilm degradation studies with r-SpeB, biofilms were left untreated for 24 h and then rinsed with PBS, and fresh TSB+ containing r-SpeB was applied. Biofilms were incubated for an additional 24 h.

### (ii) Biofilm studies with GAS strains.

For biofilm assays with GAS supernatants, AP53CovS+ and AP53CovS+ Δ*speB* strains were grown to stationary phase in THY. Cultures were spun at 3,500 rpm for 5 min to pellet bacteria, and the supernatant was removed and filtered using a 0.22-μm filter. Specified percent volumes of GAS supernatants were added to dilute USA300 cultures or preformed biofilms as described for the r-SpeB studies.

### (iii) Biofilm coculture studies.

For coculture Transwell assays, USA300 biofilms were grown untreated for 24 h before being rinsed with PBS and placed in a new 6-well plate in THY medium on the underside of a 0.4-μm Transwell membrane (Corning). GAS (AP53CovS+ and AP53CovS+ Δ*speB*) cultures (1 × 10^6^ organisms) were added to the top of the Transwell in THY and left to grow for 16 h at 37°C.

### (iv) Biofilm studies on host cell monolayers.

HaCaTs were grown to 90% confluence in DMEM supplemented with 10% FBS. The DMEM was removed and the cells were fixed using 4% paraformaldehyde (PFA) solution in phosphate buffer for 2 h at room temperature. The cells were washed five times for 5 min each in PBS before biofilm treatments were applied. For these studies, dilute overnight cultures of S. aureus (1:100) were mixed with 10% (by volume) filtered, concentrated culture supernatants from AP53CovS+ and AP53CovS+ Δ*speB*. The biofilms were grown for 24 h at 37°C prior to analysis. THY medium was used as a vehicle control for GAS treatments.

### (v) Assessment of biofilms by crystal violet assay.

For all biofilm experiments, at the end of the incubation time the supernatant was removed, the coverslip or cell monolayer was washed twice with PBS, and 0.1% crystal violet solution was applied and incubated for 15 min. The crystal violet solution was removed, and then the coverslips or cell monolayers were washed with PBS and allowed to dry. The crystal violet was resuspended in 30% acetic acid, and absorbance was measured on a microtiter plate reader at 550 nm to assess the biofilm formation or degradation.

### Live imaging of USA300 biofilms incubated with SpeB protease.

USA300 biofilms were grown untreated for 24 h on imaging dishes (MaTek) in TSB+ before being washed and incubated with PBS plus SpeB or PBS alone for 6 h. The biofilm was incubated and imaged in the environmental chamber of the Eclipse Ti-E inverted microscope (Nikon). Images were taken every 10 min for 6 h using the differential interference contrast (DIC) setting at a magnification of ×60, and video and static images were processed using ImageJ.

### Degradation of r-SdrC-A by r-SpeB *in vitro*.

Purified r-SdrC-A protein (10 μM) was incubated with r-SpeB (100 nM) at a 100:1 ratio for a time course ranging from 0 to 120 min. SdrC-A was incubated with SpeB[C^192^S] protein for the same time course as a control. Normalized amounts of protein were boiled with SDS for denaturation and loaded into 4-to-15% SDS-PAGE gradient gels. The gels were stained with Coomassie blue and destained overnight. Protein bands were imaged on the Azure Biosystem c500 system.

### Generation of rabbit polyclonal antibody against SdrC-A.

In order to generate polyclonal antibody against SdrC-A for use in our studies, New Zealand White rabbits were injected with filter sterilized r-SdrC-A and Freund’s adjuvant at the Freimann Life Sciences Center (University of Notre Dame) under the IACUC protocol 17-06-3956. Three booster injections were given at 3, 6, and 9 weeks, with a terminal bleed at 11 weeks. Rabbit serum antibody was used for our Western blot studies at a 1:1,000 dilution in blocking buffer (see “Western blotting of S. aureus biofilm degradation products”).

### Western blotting of S. aureus biofilm degradation products.

S. aureus USA300 biofilms were grown untreated on PVC coverslips for 24 h in TSB+ broth. The biofilms were washed twice with PBS to remove planktonic bacteria, then 100 nM r-SpeB or SpeB[C^192^S] in PBS was applied, and the biofilms were incubated for 2 h. Biofilms treated without protease were used as an additional control. Protein levels were normalized by *A*_280_ levels, and equal amounts of protein were run on SDS-PAGE as described above. Proteins were transferred to a polyvinylidene difluoride (PVDF) membrane overnight at 20 V. The membrane was blocked with 5% milk for 1 h, and rabbit serum primary antibody was applied for 2 h at a 1:1,000 dilution. The membrane was washed, and horseradish peroxidase (HRP)-conjugated anti-rabbit secondary antibody in blocking buffer (1:5,000 dilution) was applied to the PVDF membrane for 1 h. The membrane was washed and imaged using the chemiluminescence reagent Lumiglow (KPL).

### Mass spectrometry. (i) MALDI-TOF mass spectrometry.

MALDI-time of flight (TOF) mass spectra were acquired for positive ions using a Bruker UltrafleXtreme instrument equipped with an Nd:YAG laser operating at a repetition rates between 50 and 200 Hz for linear-mode acquisition of protein mass spectra and between 200 to 2,000 Hz for reflectron-mode acquisition of peptide mass spectra. A supersaturated solution of sinapinic acid in 50:50 water-acetonitrile with 0.5% trifluoroacetic acid was chosen as the matrix for proteins with molecular masses greater than 10,000 Da, while a supersaturated solution of 2,5-dihydroxybenzoic acid in 50:50 water-acetonitrile with 0.5% trifluoroacetic acid was chosen as the matrix for peptides with molecular weights less than 10,000 Da. MALDI-TOF mass spectra represent the summation of 1,000 to 10,000 laser shots.

### (ii) Liquid chromatography-mass spectrometry.

The LC/MS instrument consisted of a Dionex Ultimate 3000 rapid-separation ultraperformance LC system equipped with a Dionex Ultimate 3000 autosampler and a Dionex Ultimate 3000 photodiode array detector coupled with a Bruker MicrOTOF-Q II quadrupole time of flight hybrid mass spectrometer using Hystar 3.2 SR4 software. The Bruker electrospray ionization source was operated in the positive ion mode with the following parameters: end plate offset voltage of −500 V, capillary voltage of 2,000 V, and nitrogen as both a nebulizer (4 × 10^5^ Pa) and dry gas (8 liters/min) at 180°C. Mass spectra were accumulated over the mass range 500 to 6,000 Da at an acquisition rate of 5,000 per second. LC separations were performed on an Agilent Poroshell 300SB-C3 column (5 μm, 2.1-mm inside diameter by 75 mm) at 50°C. The mobile-phase (A, 0.1% formic acid in water; B, 0.1% formic acid in acetonitrile) gradient consisted of elution at 0.4 ml/min with 95% A–5% B for 3 min, followed by a 7.9-min linear gradient to 10% A–90% B, 10% A–90% B for 2 min, a 0.1-min linear gradient to 95% A–5% B, and then 95% A–5% B for 2 min. Multiply charged ions were deconvoluted using the maximum-entropy algorithm.

### Statistical analysis.

Statistical analysis was conducted using GraphPad Prism software. Student's *t* test was used for comparison of two groups, and analysis of variance was used for more than two groups. A *P* value of <0.05 was considered significant.

## Supplementary Material

Supplemental file 1

Supplemental file 2

Supplemental file 3
