# Supplementary material for: The Streptococcal Protease SpeB Antagonizes the Biofilms of the Human Pathogen Staphylococcus aureus USA300 through Cleavage of the Staphylococcal SdrC Protein
Source: J Bacteriol. 2020 May 11;202(11):e00008-20. doi: 10.1128/JB.00008-20 (PMC7221255; doi:10.1128/JB.00008-20)
Supplement: Supplemental file 1 [file JB.00008-20-s0003.pdf]

Figure S1.

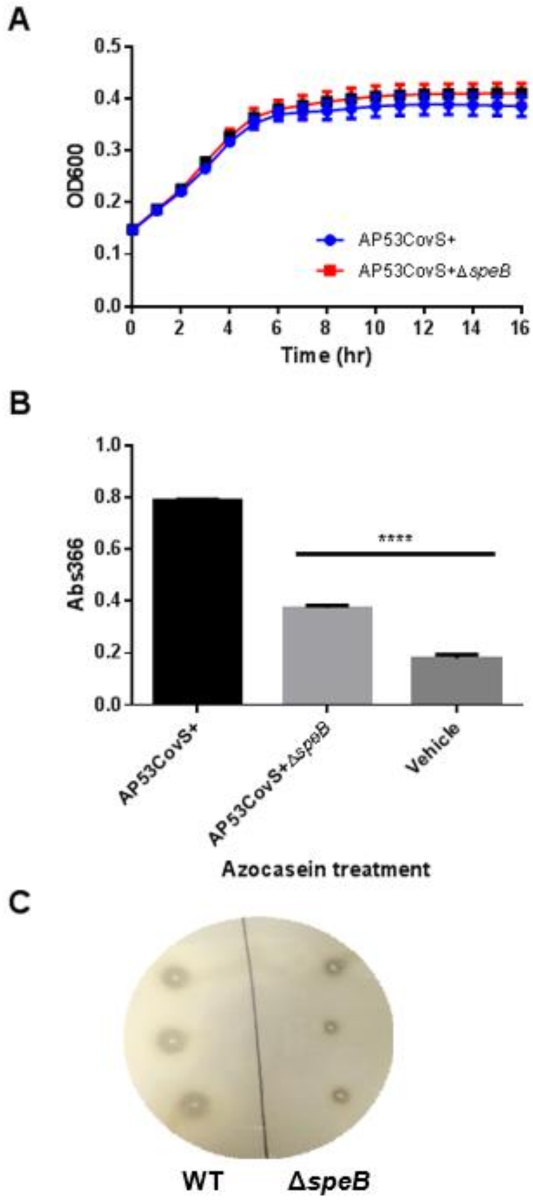

Figure S1. Characterization of AP53CovS+ GAS strains. (A). Growth curve of GAS strains AP53CovS+ and AP53CovS+Δ*speB* over 16 hours. (B). Proteolytic activity of GAS strain supernatants measured by azocasein digest. Vehicle control is THY media. (C). Zones of clearing around stabs of GAS strains in milk agar as visualization of casein digest by AP53CovS+ (WT, left) and AP53CovS+Δ*speB* (right).

Figure S2.

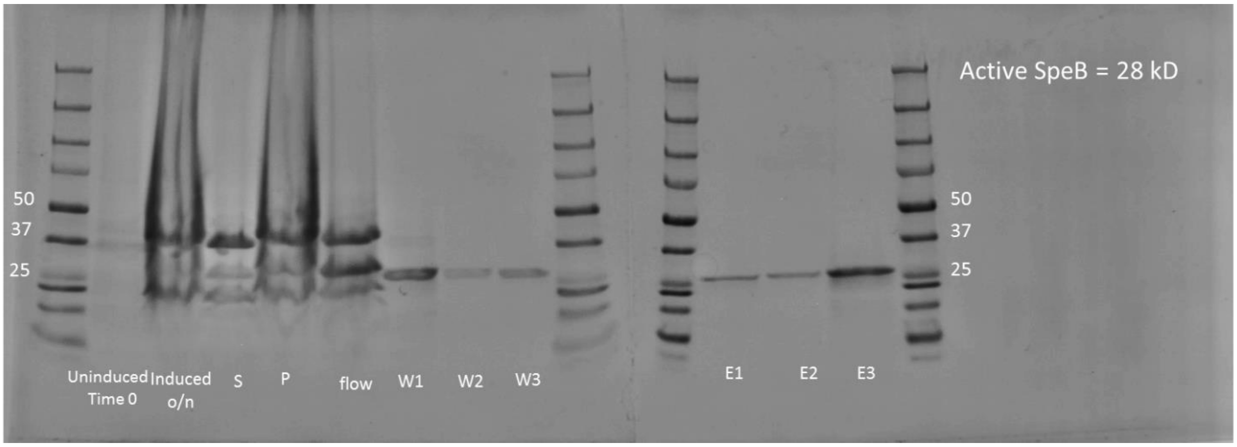

Figure S2. Purification of recombinant SpeB. The speB gene was cloned into pET42a plasmid with a C-terminal His tag. Expression was induced in BL-21 cells with IPTG, and the soluble fraction of the cell lysate was purified on a Ni-NTA column. Fractions were run on SDS-PAGE. S = souble fraction P = pellet fraction W = washes E = elutions

Figure S3.

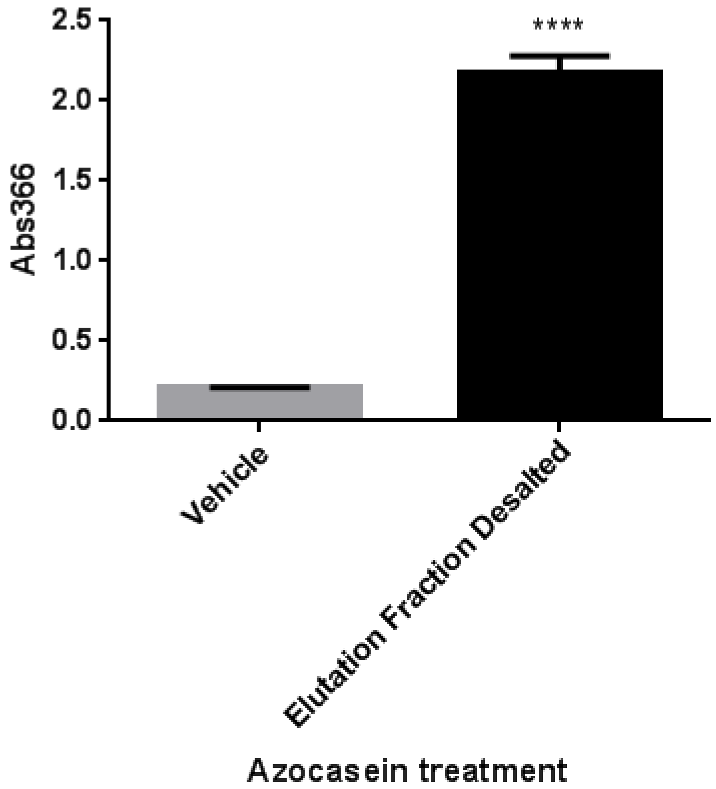

Figure S3. Azocasein digest of elution fraction from r-SpeB purification. Purified protein was incubated with azocasein reagent, and color change in the supernatant was read at 366 nm as compared to a vehicle control of elution buffer.

Figure S4.

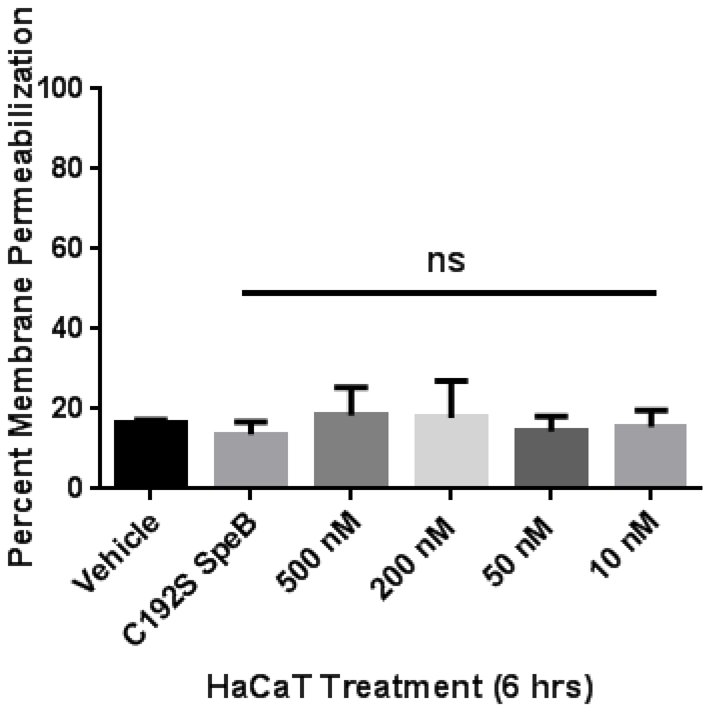

Figure S4. Cytotoxicity of r-SpeB on human keratinocytes (HaCaTs). HaCaT cells were incubated with r-SpeB and cytotoxicity was assayed by ethidium homodimer uptake after 6 hours to assess membrane permeabilization. Vehicle control of PBS was used to assess baseline cytotoxicity.

Figure S5.

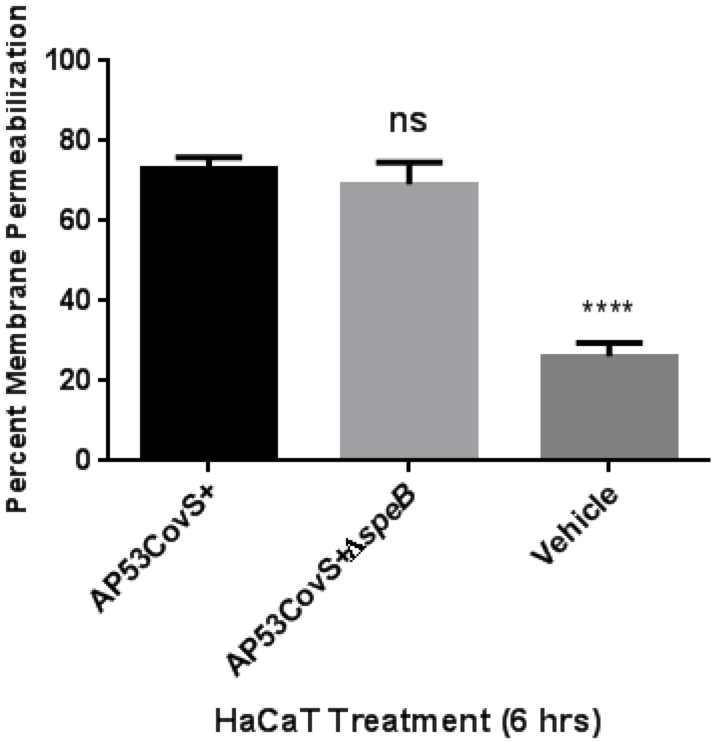

Figure S5. Cytotoxicity of AP53CovS+ and AP53CovS+Δ*speB* infection. HaCaT cells were incubated with GAS strains at an MOI of 10 bacteria per host cell and cytotoxicity was assayed by ethidium homodimer uptake after 6 hours to assess membrane permeabilization. Vehicle control of THY media was used to assess baseline cytotoxicity.

Figure S6.

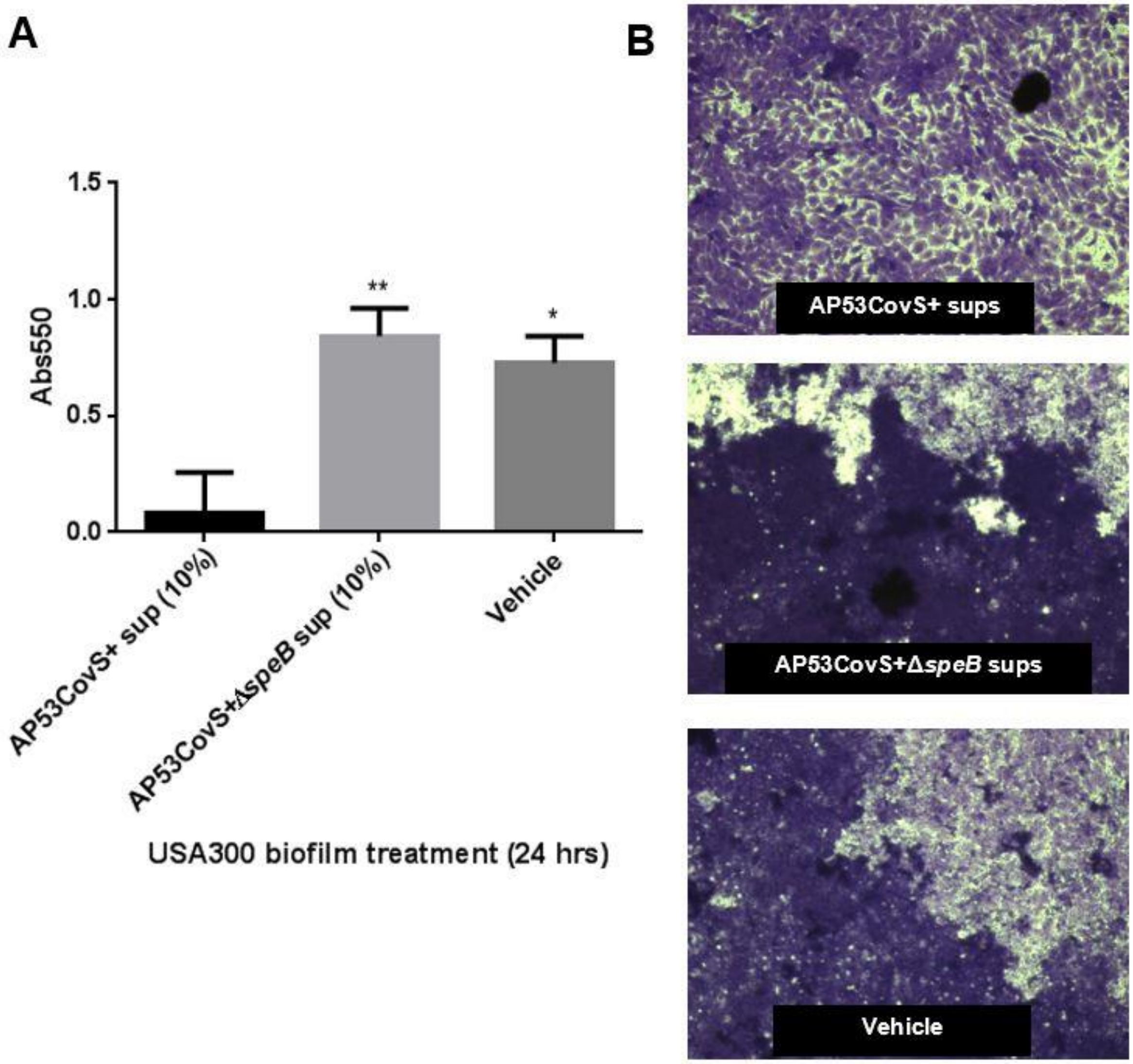

Figure S6. SpeB-mediated disruption of *S. aureus* biofilm formation on HaCaT cells. HaCaT cells were fixed in 4% paraformaldehyde prior to the addition of *S. aureus* cultures treated with GAS culture supernatants. (A). Quantification of crystal violet staining of *S. aureus* biofilms on HaCaT monolayer following 24 hours of GAS supernatant treatment. Absorbance values were normalized to crystal violet staining of uninfected HaCaT cells. **p<.01 *p<.05 (B). Representative images of crystal violet

stained HaCaTs with *S. aureus* biofilms after 24-hour treatment with AP53CovS+ supernatant (top), AP53CovS+Δ*speB* supernatant (middle), or media vehicle with no GAS supernatant (bottom).

Video S1. USA300 biofilms treated with 100 nM r-SpeB for 6 hours in PBS. Images were taken every 10 minutes using differential interference contrast (DIC)

Video S2. USA300 biofilms treated with media vehicle for 6 hours PBS. Images were taken every 10 minutes using differential interference contrast (DIC)

Figure S7.

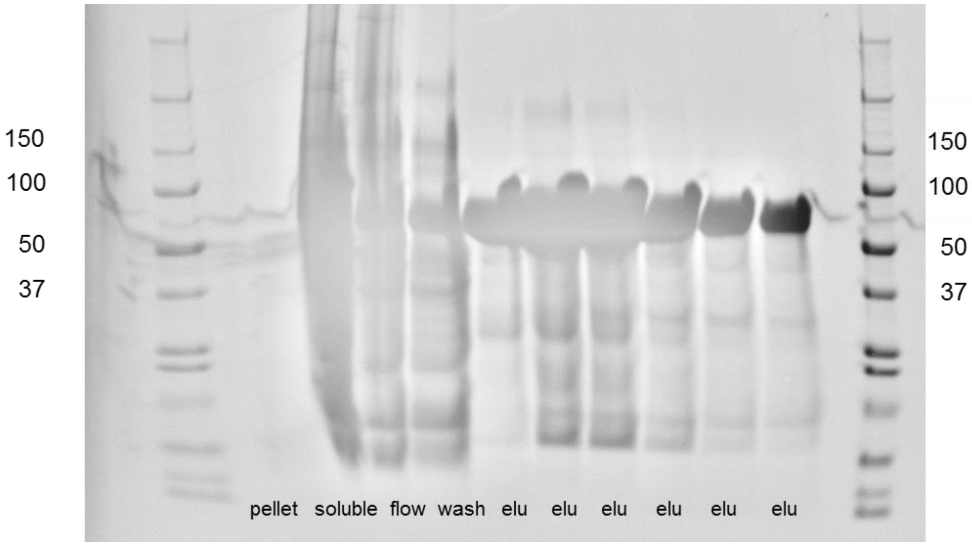

Figure S7. Purification of recombinant SdrC A Region. The SdrC gene corresponding to the A region, minus the signal sequence, was cloned into pET15 with a N-terminal His tag. Expression was induced in BL-21 cells with IPTG, and the soluble fraction of the cell lysate was purified on a Ni-NTA column. Fractions were run on SDS-PAGE. elu = elution fraction

Figure S8.

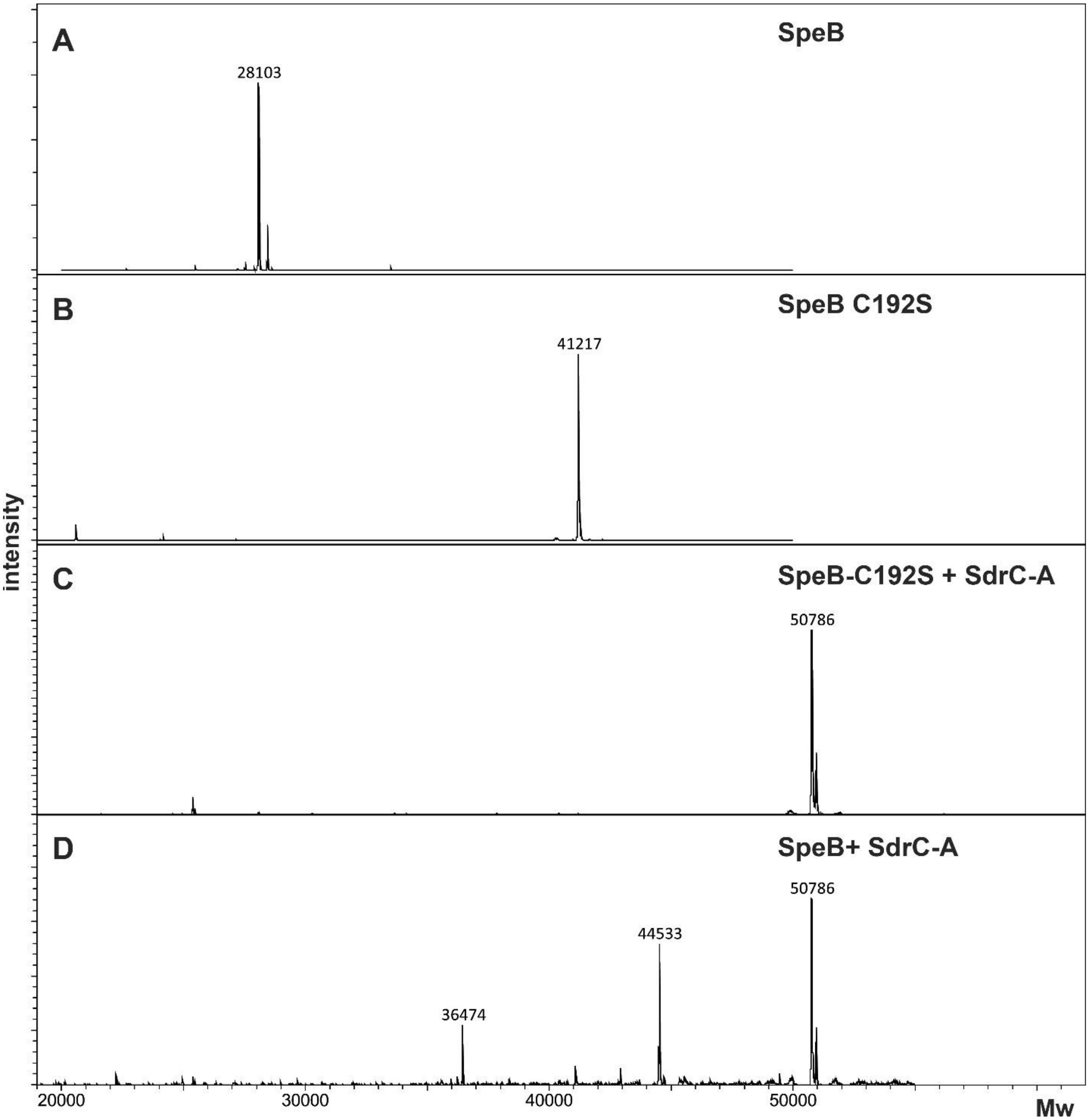

Figure S8. Deconvoluted mass spectra of (A) SpeB, of (B) SpeB[C^{192}S], of reactions of SprC-A catalyzed by (C) SpeB[C^{192}S] and by (D) SpeB from LC/MS analysis

Table S1. Primer sets used for gene deletions and expression constructs

Primer Name	Sequence
SpeBKONot5F	5’-GCGGCCGCTGAAATGAGCATCTACTAGCCAC-3’
SpeBKONot5R	5’-GGTTCTGTTCTAACGAAATGCATGCTTTTAACGGTACATTGGAC-3’
SpeBKOXho5F	5’- GTCCAATGTACCGTTAAAAGCATGCATTTCGTTAGAACAGAACC-3’
SpeBKOXho3R	5’- CTCGAGCGATGGAAACAGTCTGCTCCATG-3’
SpeBext5F	5’- AGCAGCTATGATATAGCCATAAGG-3’
SpeBext3R	5’- GTCATCATTAGTAGGCGTTGATG-3’
SpeB-F	5'-GCGCATGATCCATATGGATCAAAACTTTGCTCGT-3’
SpeB-R	5'-GCGCATGATCGAATTCTCAGTGGTGGTGGTGGTGGTGAGGTTTGAT GCCTACAACAGC-3’
P1, speB5FNdeI	5’-GCGCATGATCCATATGGATCAAAACTTTGCTCGT-3’
P2, speB3RC192S	5’-AGCAGTTGCAGTAGCAACAgATCCTGTAGCTGCATGTTG-3’
P3, speB5FC192S	5’-CAACATGCAGCTACAGGATcTGTTGCTACTGCAACTGCT-3’
P4, speB3REcoRI	5’-GCGCATGATCGAATTCTCAGTGGTGGTGGTGGTGGTGAGGTTTGAT GCCTACAACAGC-3’
pET15_sdrC-F	5’-TCATCATCATCATATGGAACATACGAATGGAGAATTAAATC-3’
pET15_sdrC-A-R	5’-TATGCTAGTTACTAGTTTATTTCTTTTGGTCGCCATTAGC-3’
OMF512	5’-ACTAGTAACTAGCATAACCCCTTGGGGC-3’
OMF513	5’-CATATGATGATGATGATGATGGCTGC-3’
